# Setdb1, a novel interactor of ΔNp63, is involved in breast tumorigenesis

**DOI:** 10.18632/oncotarget.7089

**Published:** 2016-01-31

**Authors:** Carla Regina, Mirco Compagnone, Angelo Peschiaroli, AnnaMaria Lena, Margherita Annicchiarico-Petruzzelli, Maria Cristina Piro, Gerry Melino, Eleonora Candi

**Affiliations:** ^1^ Department of Experimental Medicine and Surgery, University of Rome “Tor Vergata”, Rome, Italy; ^2^ CNR, Institute of Cell Biology and Neurobiology (IBCN), Rome, Italy; ^3^ IDI-IRCCS, Rome, Italy

**Keywords:** p63, SETDB1, breast cancer, histone methyl transferase, proliferation

## Abstract

ΔNp63 has been recently involved in self-renewal potential of breast cancer stem cells. Although the p63 transcriptional profile has been extensively characterized, our knowledge of the p63-binding partners potentially involved in the regulation of breast tumour progression is limited. Here, we performed the yeast two hybrid approach to identify p63α interactors involved in breast tumorigenesis and we found that SETDB1, a histone lysine methyl transferases, interacts with ΔNp63α and that this interaction contributes to p63 protein stability. SETDB1 is often amplified in primary breast tumours, and its depletion confers to breast cancer cells growth disadvantage. We identified a list of thirty genes repressed by ΔNp63 in a SETDB1-dependent manner, whose expression is positively correlated to survival of breast cancer patients. These results suggest that p63 and SETDB1 expression, together with the repressed genes, may have diagnostic and prognostic potential.

## INTRODUCTION

p63, member of the p53 family, is a master regulator of epithelial biology, including mammary gland, where it is indispensable to maintain the high proliferative potential of somatic and cancer stem cells [1]. The detailed mechanisms of p63 and, particularly, the relative contribution of the distinct pathways exerted by different p63 isoforms during epithelial tumorigenesis remain partially obscure at the molecular level [2, 3]. Most of this controversy is probably due to the existence of multiple isoforms with contrasting biological functions. Indeed, TP63 gene has two distinct promoters expressing proteins with distinct and often contrasting biological functions, including a full length and an amino-deleted isoform, named TAp63 and ΔNp63, respectively. TAp63 isoforms contain a canonical p53-like transactivation domain (TA) and, at the physiological level, is predominantly expressed in oocytes where it acts as the “guardian of the female germline” [4]. Conversely, ΔNp63, the shorter isoform without the N-terminal TA domain but still able to transcribe due to the presence of a second TA domain [5], is primarily expressed in the epithelial tissue [6–11]. Both TAp63 and ΔNp63 mRNAs undergo to alternative splicing at the 3′-end, to generate proteins with unique C-termini, named alpha, beta and gamma, whose biological functions have not yet been deeply studied. The alpha isoforms, compared to p53, have an extended C-terminus containing the Oligomerization Domain (OD), the SAM (Sterile Alpha Motif) domain, a protein-protein interaction domain, and the TI (Trans Inhibitory) domain.

While mutations of p63 are extremely rare in human cancers, several tumors (> 80% of primary head and neck squamous cell carcinomas [HNSCCs], squamous cell epithelial lung malignancies, and basal-like subtype of breast cancer [12–14], often display elevated levels of ΔNp63, due, in some cases, to gene amplification. Functionally, in HNSCCs, ΔNp63 acts as a potent oncogene and its acute genetic ablation determines a rapid tumor regression, suggesting the importance of this isoform in driving tumor proliferation and/or blocking apoptosis [15, 16]. Human breast cancer recent reports demonstrated the importance of ΔNp63 in promoting the tumour-initiating activity of the basal and luminal breast cancer cells. Also, different transcriptional targets of ΔNp63 have been identified, underlying its role in controlling mammary cancer stem cells homeostasis [1, 14]. Being the stemness properties of cancer cells strictly correlated with tumor aggressiveness, it is not surprising that ΔNp63 expression has been functionally associated with the deregulation of tumor invasiveness and tumor cell migration [17, 18]. All these data indicate that p63, likely ΔNp63, is an important regulator of breast tumor progression and metastasis. As stated before, ΔNp63 isoforms possess a transcriptional activation domain, which allows these proteins to act as transcriptional activators towards a subset of target genes involved in the regulation of cell proliferation, cell survival and tumour growth. However, ΔNp63 may also act as a transcriptional repressor, in fact several mechanisms, involving for instance HDAC or H2AZ interaction, have been described [15, 19].

Here, we identified by yeast two-hybrid a p63 binding protein potentially involved in the ΔNp63-mediated transcriptional repression. We found that SETDB1, a histone lysine methyl transferase, interacts exclusively with the ΔNp63 isoforms. By generating deletion mutants of ΔNp63α and SETDB1 we identified the domain of p63 and SETDB1 responsible for the interaction. We showed that SETDB1 is often amplified in primary breast tumours and it is overexpressed at the protein level in breast cancer cell lines. Functionally, SETDB1 silencing in breast cancer cells results in tumour cell growth disadvantage. We also identified a list of thirty genes repressed by ΔNp63 in a SETDB1-dependent manner, some of them positively correlated to the survival of breast cancer patients. These data suggest that p63 and SETDB1 expression, together with the repressed genes, may have diagnostic and prognostic potential.

## RESULTS

### ΔNp63α binds to SETDB1

**Figure 1:**
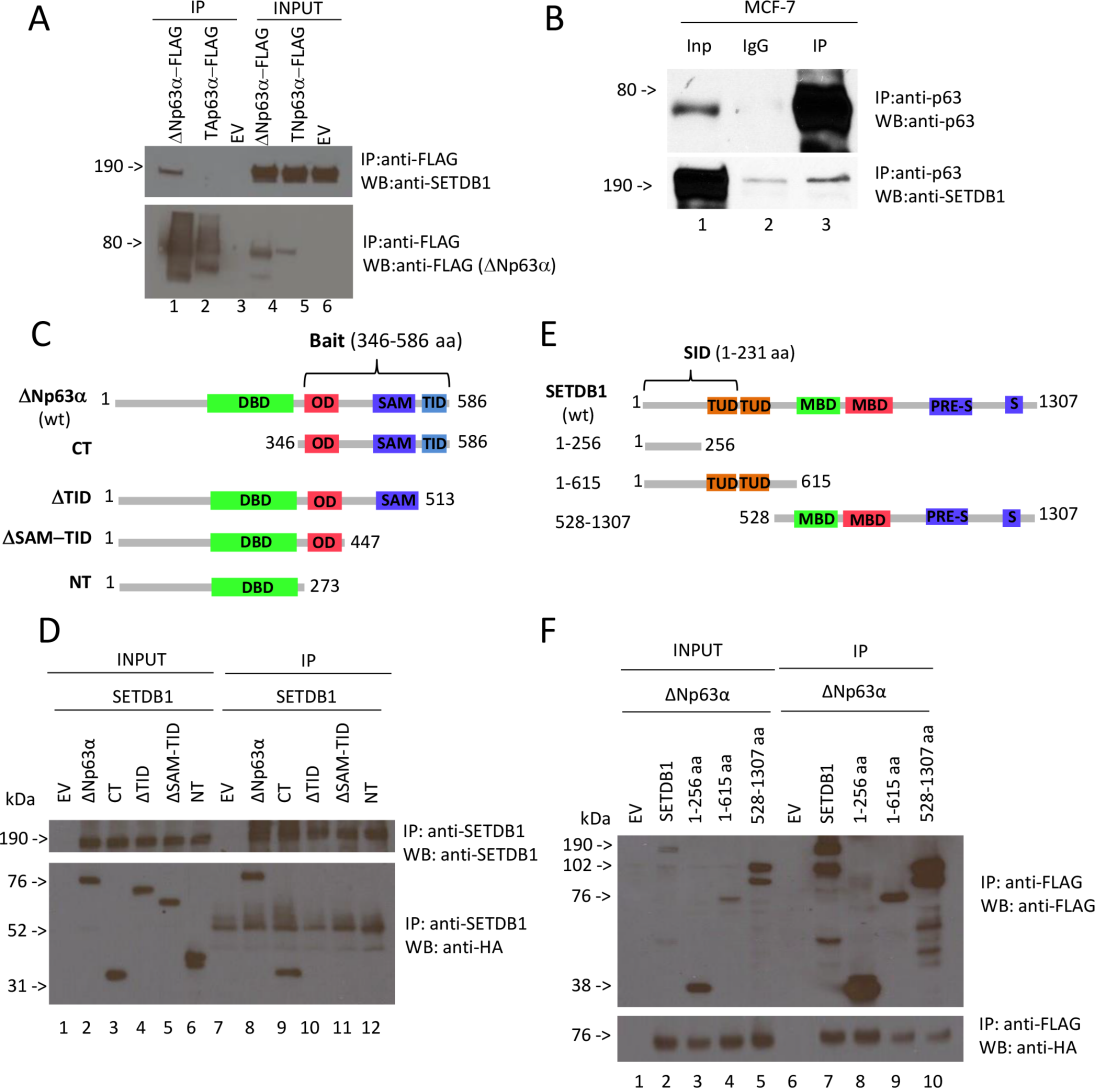
ΔNp63α binds SETDB1 (**A**) Semi-endogenous immunoprecipitation of p63α isoforms and SETDB1. Flag-TAp63α and Flag-ΔNp63α expression vectors were transiently transfected in H1299 cells. Cell extracts were immunoprecipitated with anti-Flag antibody and subjected to western blot analysis (lanes 4 to 6) with anti-SETDB1 antibody (upper panel) and anti-Flag antibody (lower panel). Aliquots of total cell extracts from unprocessed cells were also loaded on the gel (lanes from 1 to 3). EV, empty vector. (**B**) Immunoprecipitation of endogenous p63 with endogenous SETDB1. MCF7 cells extracts were immunoprecipitated with anti-p63 antibody and subjected to western blot analysis (lane 3) with anti-SETDB1 antibody (lower panel) and anti-p63 antibody (upper panel). The aliquot of total cell extract from unprocessed cells (lane 1) and IgG, used as negative control, (lane 2) were also loaded on the gel. Quantification of SETDB1 IP/SETDB1 IgG = 1,8 fold. (**C**) It is shown wt ΔNp63α containing all domains of the protein: Transactivation domain of ΔN isoforms (TA1, not shown), DNA-binding domain (DBD), oligomerization domain (OD), transactivation domain 2 (TA2, not shown), sterile alpha motif (SAM) and transactivation inhibitory domain (TID); the first mutant contains only the C-terminus including OD, TA2 (not shown), SAM, TID (CT); the second one contains all domains apart from TID (ΔTID); the third one contains all domains apart from SAM and TID (ΔSAM-TID); the fourth one contains all domains apart from OD, TA2, SAM, TID (NT). (**D**) Coimmunoprecipitation of p63 deletion mutants and SETDB1. HA-ΔNp63α, HA-CT, HA-ΔTID, HA- ΔSAM-TID, HA-NT expression vectors were transiently transfected in H1299 cells together with full lenght Flag-SETDB1. Cell extracts were immunoprecipitated with anti-SETDB1 antibody and subjected to western blot analysis (lanes 7 to 12) with anti-SETDB1 antibody (upper panel) and anti-HA antibody (lower panel). Aliquots of total cell extracts from unprocessed cells were also loaded on the gel (lanes 1 to 6). EV, empty vector; NT, no transfection. (**E**) It is shown wt SETDB1 containing all domains of the protein: Tudor domains (TUD), methyl-CpG-binding domain (MBD), PRE-SET domain and the bifurcated SET domain (S-ET). Post-SET domain is not shown; the first mutant (1-256 aa) contains only the N-terminus, lacking all functional domains; the second mutant (1- 615 aa) contains the N-terminus and both Tudor domains; the third mutant contains MBD (methyl-CpG-binding domain), PRE-SET and the bifurcated SET domain. (**F**) Coimmunoprecipitation of SETDB1 deletion mutants and ΔNp63α. Full lenght Flag-SETDB1, Flag-1-256aa, Flag-1-615aa and Flag-528-1307aa expression vectors were transiently transfected in H1299 cells together with full lenght ΔNp63α. Cell extracts were immunoprecipitated with anti-Flag antibody and subjected to western blot analysis (lanes 6 to 10) with anti-Flag antibody (upper panel) and anti-HA antibody (lower panel). Aliquots of total cell extracts from unprocessed cells were also loaded on the gel (lanes 1 to 5). EV, empty vector. Uncropped images of gels are shown in Supplementary Figure S5.

In order to identify proteins that are able to interact with p63 and regulate its function in breast cancer, we performed a yeast two-hybrid screening using as bait the C-terminal fragment of ΔNp63α (amino acids 346 to 586), which contains the OD, the SAM and the TI domains (Figure 1). As prey we utilized the Human Breast Tumor Epithelial cDNA library. We identified several clones containing the SETDB1 N-terminal fragment (amino acid 1–231) as selected interaction domain (SID) (Figure 1), indicating that the interaction may occur between the p63 C-terminus and the SETDB1 N-terminus. We firstly confirmed the interaction between p63 and SETDB1 at semi-endogenous level (Figure 1A). H1299 cells were transfected with Flag-tagged TAp63α and ΔNp63α and then the exogenous proteins were immunoprecipitated to evaluate their interaction with endogenous SETDB1. As shown in Figure 1A, SETDB1 interacts specifically with ΔNp63α. We also confirmed the interaction between SETDB1 and ΔNp63α at endogenous level in MCF- 7 breast cancer cell line, which express exclusively the ΔNp63α isoform (Figure 1B lane 3; Supplementary Figure S1A–S1D), and in normal human epidermal keratinocytes (Supplementary Figure S2).

In order to map ΔNp63α domains responsible for SETDB1 binding, we generated HA-tagged ΔNp63α deletion mutants and performed co-immunoprecipitation experiments in SETDB1 overexpressing H1299 cells (Figure 1C, 1E). We immunoprecipitated exogenous SETDB1 using an anti-SETDB1 antibody and then we stained the immunocomplexes with anti-HA antibody. As shown in Figure 1D, the full length ΔNp63α interacts with SETDB1 (Figure 1D, lane 8) as well as the CT mutant (lane 9). The deletion of the TID domain abrogates the p63 binding to SETDB1 indicating that this C-terminal domain is necessary for the interaction (Figure 1D, lanes 10–12). In parallel, we also mapped the protein regions of SETDB1 responsible for ΔNp63α binding. FLAG-tagged SETDB1 deletion mutants and HA-tagged ΔNp63α were co-transfected in H1299 cells and then immunoprecipitated with anti-Flag antibody. As shown in Figure 1F all SETDB1 mutants interact with ΔNp63α (Figure 1F), suggesting that both SETDB1 N- and C-termini are involved in the interaction. Our results clearly demonstrated, for the first time, that ΔNp63α physically interacts with SETDB1.

### SETBD1 affects p63 protein level

**Figure 2:**
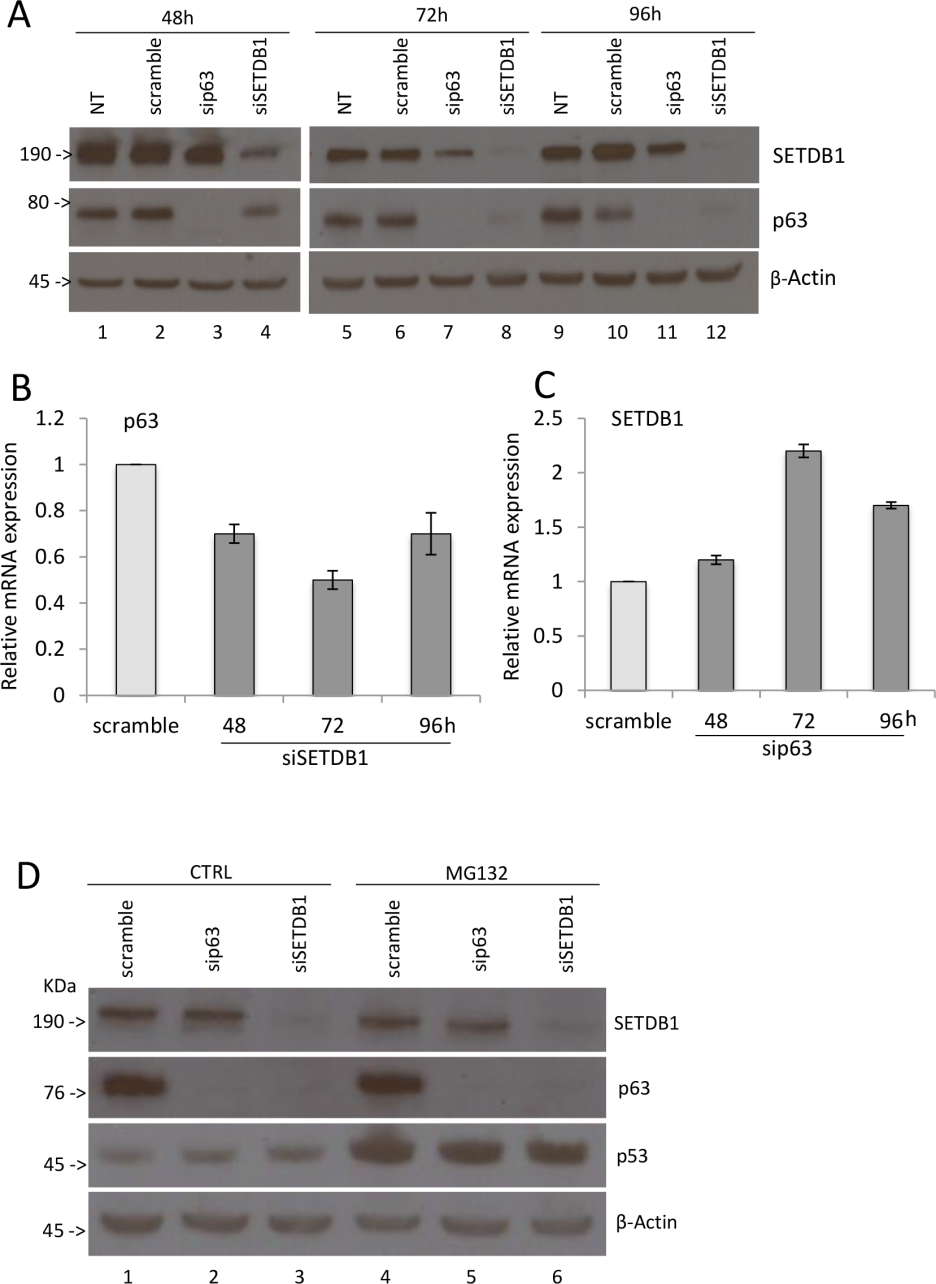
SETDB1 affects p63 protein stability (**A**) Western blot showing p63 and SETDB1 protein expression after transient silencing of p63 or SETDB1 in MCF7 cell line. Cells were collected 48 hours, 72 hours, 96 hours after the silencing. β-Actin is shown as loading control. NT, no transfection. One representative experiment of three is shown. (**B**) Relative quantification of p63 mRNA after SETDB1 transient silencing (48-72-96 hours). One representative experiments of three is shown. (**C**) Relative quantification of SETDB1 mRNA after p63 transient silencing (48-72-96 hours). One representative experiments of three is shown. Uncropped images of gels are shown in Supplementary Figure S6. (**D**) Western blot showing p63 and SETDB1 protein expression after transient silencing of p63 or SETDB1 in MCF7 cell line. Cells were collected 72 hours after the silencing and treatment with MG132. β-Actin is shown as loading control. One representative experiment of three is shown. Uncropped images of gels are shown in Supplementary Figure S7.

In order to verify if this interaction affects ΔNp63α and/or SETDB1 protein levels, we silenced p63 or SETDB1 expression by siRNA in MCF-7 cells and performed immunoblotting analysis to evaluate their protein levels. As shown in Figure 2A, we observed that SETDB1 depletion strongly reduces p63 expression, which is almost undetectable at 72 and 96 hours post siSETDB1 transfection (compare lanes 2 and 4, lanes 6 and 8, lanes 10 and 12). At a less extent, silencing of p63 reduced SETDB1 expression after 48 and 72 hours (compare lanes 6 and 7; 10 and 11). To evaluate if this reduction is due to inhibition of transcription, we evaluated p63 and SETDB1 mRNA levels upon silencing. As shown in Figure 2B, p63 mRNA level is reduced to 55–70% upon SETDB1 depletion, indicating the p63 reduction at protein level might be due to transcriptional inhibition. On the contrary, p63 depletion did not affect SETDB1 mRNA levels (Figure 2C). Interestingly, the treatment with the proteasome inhibitor MG132 did not restore p63 protein levels upon SETDB1 silencing (Figure 2D), suggesting that other mechanisms, possibly microRNAs, may be involved in their reciprocal regulation.

These results indicate that the binding of SETDB1 to p63 contributes to stabilize p63 protein levels in breast cancer cells.

### Expression of SETDB1 and its growth-promoting effects in breast cancer cell lines

**Figure 3:**
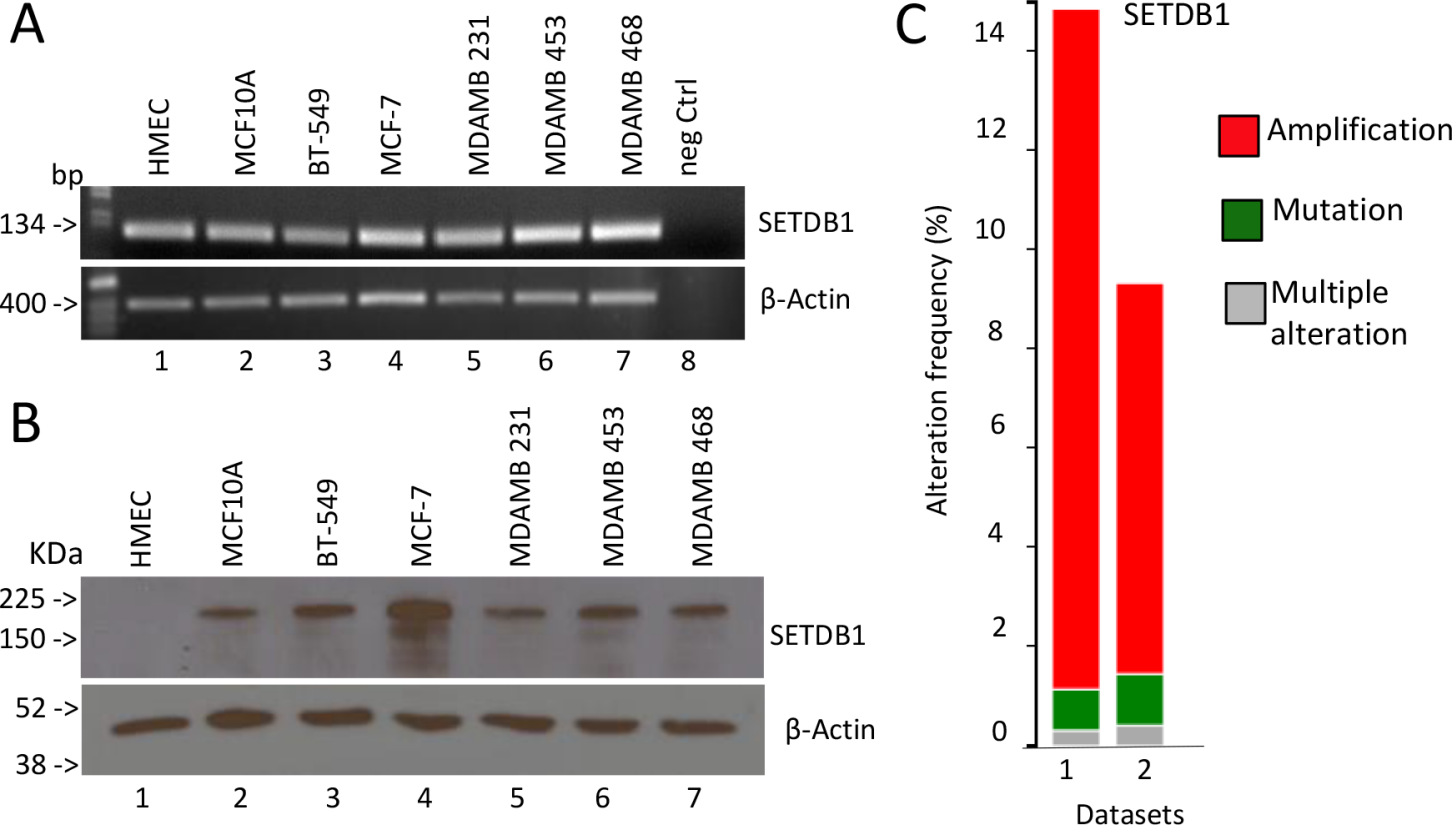
Expression analysis of SETDB1 in breast cancer (**A**) Semi-quantitative reverse transcriptase analysis of SETDB1 mRNA levels in different breast cancer cell lines (basal type: BT-549, MDM-MB231, MDM-MB468; luminal type: MCF-7, MDM-MB453). Human Mammary Epithelial Cells (HMEC) and MCF-10A have been used as normal primary and immortalized breast epithelial cells). β-Actin is shown as loading control. One representative experiments of three is shown. (**B**) Western blot analysis of SETDB1 in the breast cell lines stated above. β-Actin is shown as loading control. One representative experiments of three is shown. Uncropped images of gels are shown in Supplementary Figure S8. (**C**) High level of amplification of the histone methyl transferase, SETDB1, in breast cancer datasets obtained from The Cancer Genome Atlas, via cBioPortal.

It is known that alteration of histone modification landscape, which is controlled also by histone lysine methylation, is a common event in cancer cells. To determine whether SETDB1 expression is altered in breast tumour cells, we measured SETDB1 mRNA and protein levels in basal-type (BT-549, MDM-MB231, MDM-MB468 and HCC1954), luminal-type (MCF-7, MDM-MB453), non tumorigenic immortalized breast epithelial (MCF-10-A) cell line and in the normal mammary epithelial cells (HMEC). As shown in Figure 3A, SETDB1 is detected at mRNA level by semi-quantitative RT-PCR in all cell lines tested. Interestingly, SETDB1 protein level is not detected in normal mammary epithelial cells (HMEC), indicating that post-transcriptional mechanisms contribute to SETDB1 accumulation in breast cancer cell lines (Figure 3B). To determine whether breast cancer primary human tumours display increased levels of SETDB1, we performed a bioinformatic analysis of SETDB1 copy numbers and mutations using the cBio Cancer Genomics Portal (http://www.cbioportal.org). We analysed two breast carcinoma datasets and we found that in TCGA-Provisional and TCGA-Nature 2012 datasets SETDB1 is amplified in 13.7% and 7, 9% of patients, respectively (Figure 3C) [21, 22].

**Figure 4:**
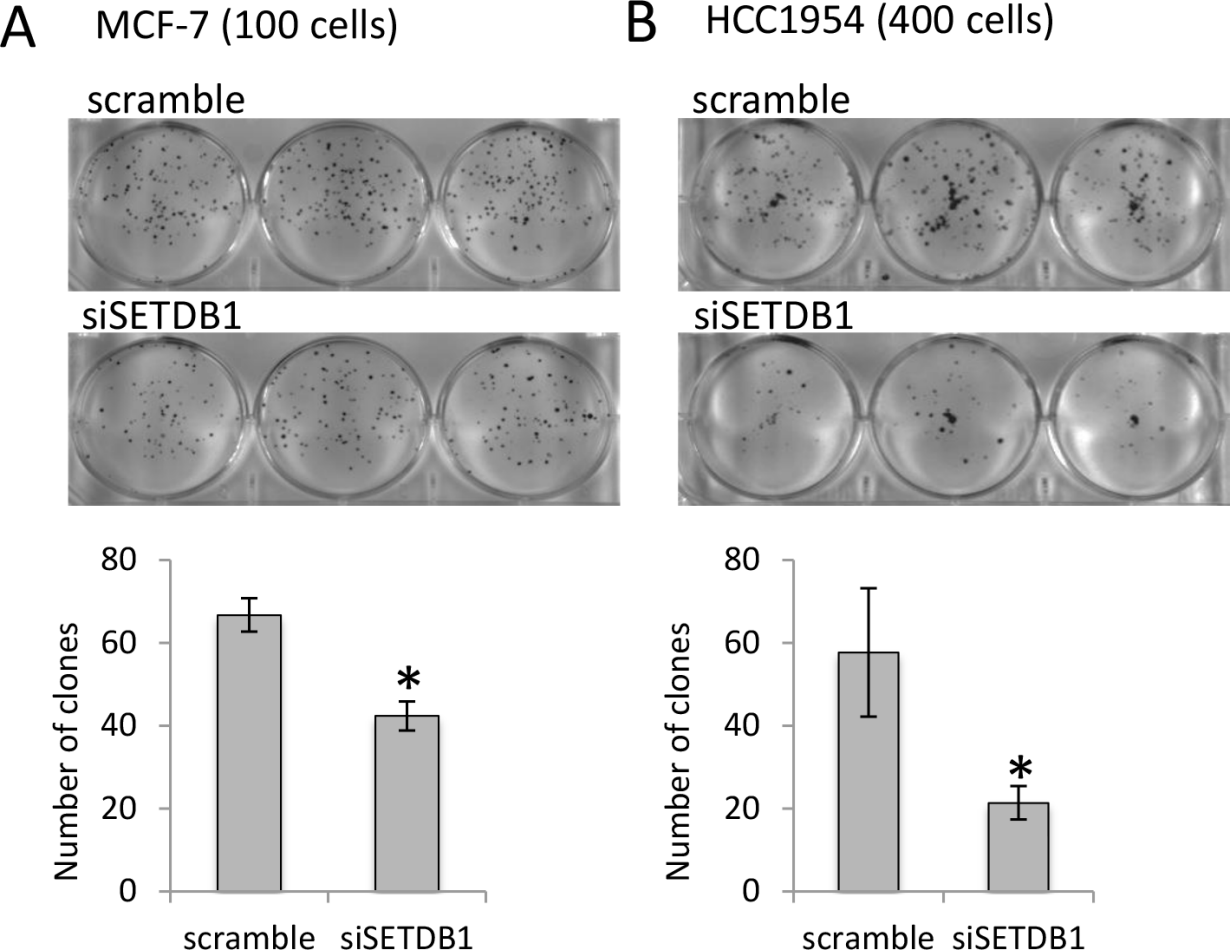
SETDB1 growth-promoting effects in breast cancer cell lines (**A**) MCF-7 colony formation assay (by counting 100 cells plated) comparing si-SETDB1 cells with si-scramble cells. One representative experiment of three is shown. Graphs represent quantification. (**B**) HCC-1954 colony formation assay (by counting 400 cells plated) comparing si-SETDB1 cells with si-scramble cells. One representative experiment of three is shown. Graphs represent quantification.

Having determined that SETDB1 is over-expressed in breast cancer cell lines, we evaluated its functional contribution to the tumor cell growth *in vitro* by performing a colony forming assay in HCC1954 and MCF-7 breast cancer cells (Figure 4A–4B) depleted of the expression of SETDB1. We found that the reduction of SETDB1 expression significantly reduces the colonies number to 46% and 19% in MCF-7 and HCC1954, respectively (Figure 4). Interestingly, depletion of SETDB1 also caused induction of cell death (four fold increases, Supplementary Figure S3A–S3B) and reduction of proliferation (Supplementary Figure S3A–S3C) in HCC1954. These data indicated that the increased expression of SETDB1 in primary breast cancer tumours and in the breast cancel cell lines is functional to enhance tumour cell growth by sustaining tumor cell proliferation and survival. To determine if ΔNp63-SETDB1 interaction has effects on H3K9me3 deposition, we performed confocal and western blot analysis in p63 depleted cells to detect H3K9me3 using a specific anti-H3K9me3 antibody. Results showed a reduction of H3K9me3 mark (30% reduction evaluated by densitometry quantification; Supplementary Figure S4A–S4B) in p63 depleted cells, suggesting that ΔNp63α, likely by interacting with SETDB1, contributes to the H3K9me3 deposition.

### Genes repressed by p63 in a SETBD1-dependent fashion

**Figure 5:**
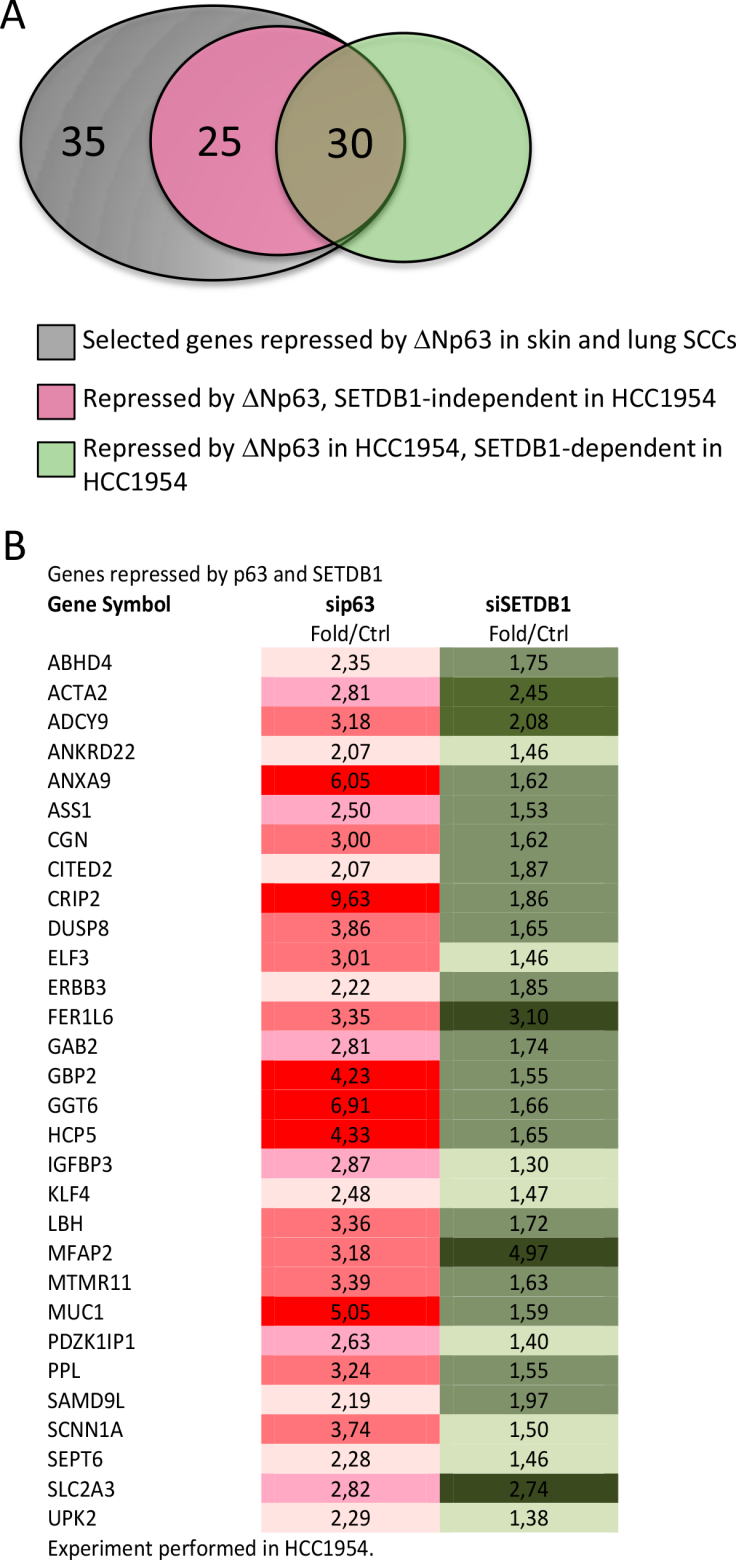
Genes repressed by both p63 and SETDB1 in HCC1954 (**A**) Venn diagram indicating that of the 90 selected genes repressed by ΔNp63 in skin e lung SCCs, 35 genes are not repressed in HCC1954, 25 are repressed in a SETDB1-independent fashion and 30 are repressed in SETDB1-dependent fashion. (**B**) List of 30 genes repressed both by p63 and SETDB1. The data are shown are fold over control.

**Figure 6:**
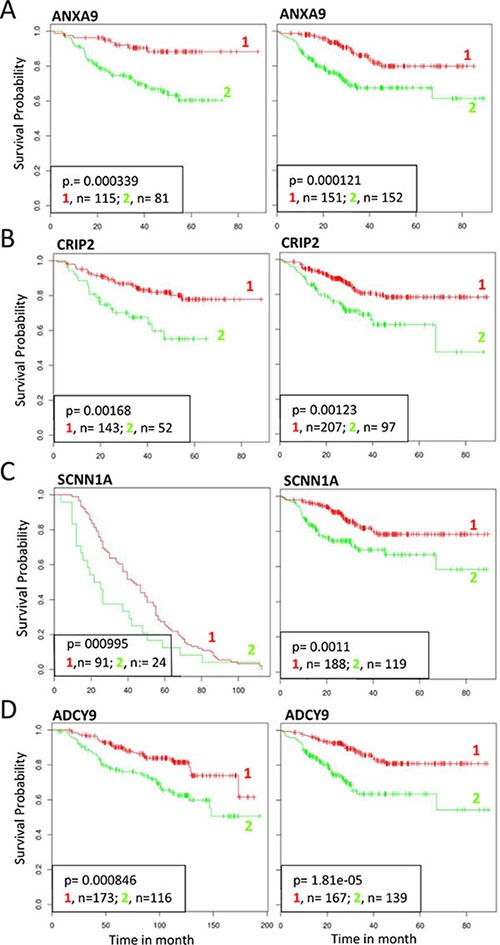
Survival analysis of selected genes repressed by p63 and SETDB1 Effect on survival outcome of selected genes (ANXA2 CRIP2 SCNN1A ADCY9) repressed both by p63 and SETDB1. Clinical follow up data of different breast cancer datasets were censored for survival. Kaplan–Meier analysis showed a significant positive correlation with survival in two datasets. 1, RED: high expression; 2, GREEN, low expression. The following datasets were used: (**A**) ID:GSE25065 (left), ID:GSE25055 (right); (**B**) GSE25065 (left), GSE25055 (right); (**C**) GSE30682 (letf), GSE25055 (right); (**D**) GSE25065 (left), GSE25055 (right) [21, 22].

Being SETDB1 an histone H3 lysine 9-specific methyltransferase component of the Polycomb repressive Complex 2, we investigated if SETDB1 may participate in the ΔNp63 repressor activity. We created a list containing 90 genes potentially repressed by ΔNp63α. This list was obtained by crossing data from previous arrays in normal human keratinocytes [9] and squamous cell carcinomas [19] with the list of genes negatively correlated with p63 expression in SCC primary tumours (cBio Cancer Genomics Portal; http://www.cbioportal.org). We analyzed the expression of the 90 selected genes after p63 and SETDB1 silencing in HCC1954 cells. Our results showed that of the 90 selected genes, 55 genes (61%) were upregulated in sip63 HCC1954 cells (Figure 5A–5B, 2 folds cutoff, red color). Thirty genes (54.5%) of the 55 were also upregulated in SETDB1 depleted cells, indicating that ΔNp63 might repress the expression of a subsets of genes in a SETDB1-dependent manner (Figure 5A–5B, cut off 1.3, green color). The expression of the rest of the genes (60 genes, 45.5%) was not altered upon SETDB1 silencing, indicating that their expression is SETDB1-independent (Supplementary Table S1). To gain further information on the functional role of p63-SETDB1 interaction in breast cancer, we performed patients survival analysis of several genes that are regulated in a SETDB1- and p63-dependent manner using GINT database (Gene Interaction survival analysis In Cancer, http://bioprofiling.de) [21, 22]. We found that the low expression of Annexin A9 (ANXA9), cysteine-rich intestinal protein 2 (CRIP2), Sodium Channel, Non-Voltage-Gated 1 Alpha Subunit (SCNN1A) and Adenylate cyclase 9 (ADCY9) is negatively correlated to patients survival in at least two breast cancer datasets Figure 6, Supplementary Table S2). These results suggest a novel molecular SETDB1-dependent mechanism potentially involved in mediating the ΔNp63 oncogenicity in breast cancer cells, and therapeutically relevant for ΔNp63α over-expressing patients. In addition, we suggest that the identified genes subset may be relevant in breast cancer as biomarkers for diagnosis and prognosis.

## DISCUSSION

TP63 is a transcription factor belonging to the p53 gene family, which includes p73 and p53 [23–25]. p53 is the best studied member of the family, showing a complex genes activation programs from DNA damage repair [26–29], stemness and lineage determination [30, 31], autophagy [32, 33], mitochondria, metabolism and ROS regulation [34–36]. Although being identified later, already now, p63 and p73 show their complexity and interaction with p53 [37–42], where p63 function is highly relevant in skin formation and homeostasis [43] as well as in cancer [40–44]. Indeed, ΔNp63 is frequently overexpressed in carcinomas of epithelial origin, including skin and lung squamous cell carcinoma (SCC) and basal breast carcinomas, where it functionally sustains tumor growth by regulating a subset of transcriptional targets. It is well accepted that ΔNp63 can be both a positive and a negative regulator of transcription, but it is unclear how these properties exactly contribute to its oncogenic potential. Furthermore, studies on the functional characterization of p63 binding partners have been neglected respect to those aimed to analyze p63-dependent transcriptional profile. Here, we performed a yeast two-hybrid experiment in order to identify p63α interactors potentially involved in breast tumorigenesis. Among them, the histone methyltransferase, SETDB1, was identified. Interestingly, SETDB1 interacts selectively with ΔNp63α but not with TAp63α, although both isoforms carry an identical C-terminal domain. This is probably due to different structures of the two isoforms as demonstrated by Dotsch's laboratory [45–47]. SETDB1 is a Histone H3 lysine 9-specific MethylTransferase (HMT) belonging to the SET (Suppression of variegation, Enhancer of zeste, Trithorax)-domain containing enzymes, important in epigenetic regulation [48]. HMTs catalyze the transfer of one to three methyl groups from S-adenosylmetionine to specific lysine residues on histone proteins [49]. Depending on the site and degree of methylation, the modification can lead to various effects including regulation of chromatin organization and gene transcription.

Alteration of chromatin state has offen been reported in cancer, however which specific chomatin regulator is involved in different cancer cells is not clear [50–52]. For example, H3K9me3 and H3K27me3 marks associated to heterochromatin repressed domains in normal cells that are misregulated in cancer [53]. The H3K9me3 is formed by a family of histone methyltransferases including SUV39H1, that have been recently described to be associated with cancer. SUV39H1 inhibition is sufficient for re-expression of the silenced tumor suppressor genes CDKN2B and CDH1 marked by H3K9me3 [54] in acute myeloid leukemia. While deposition of H3K27me3 by EZH2 enzyme is often increased in aggressive breast cancers [55–57], and mutation in the H3K27me3 demethylase KDM6A are common in renal cell carcinoma [58]. Recent studies have also shown that loss of DNA methylation in breast cancer cell line (HCC1954) is accompanied by formation of repressive chromatin, with a significant increses of histone modifications H3K9me3 or H3K27me3 [57]. Among the different HAT, SETDB1 has been of increasing interest due to its recent involvement in melanoma, where it is located in a recurrently amplified chromosome fragment [59] and in lung tumors [60]. Here, we found that SETDB1 is overexpressed at protein level in breast cancer cell lines and that its gene is amplified in primary tumours, as also confirmed in a meta-analysis study recently published [61]. Furthermore, depletion of SETDB1 results in tumour cell growth disadvantage, indicating that it possibly acts as oncogene also in breast cancer cells in combination with ΔNp63α. Our results strongly suggest that in breast cancer, ΔNp63, by physically interacting with SETDB1, could redirect SETDB1 to specific genomic regions and therefore alter H3K9me3 mark responsible for chromatin modification and gene silencing. So far, two different p63-dependent transcriptional repression mechanisms have been identified that are differently utilized in several cell types: p63 recruitment of histone deacetylases [62] and p63-dependent deposition of the histone variant H2A.Z [19]. Our data propose that an alternative third mechanism, involving the SETDB1 methyl transferase, might be utilized by ΔNp63α. This indicate that ΔNp63α utilizes multiple mechanisms of repression in a combinatorial fashion and a cell-type specific manner.

## MATERIALS AND METHODS

### Cell culture and silencing conditions

H1299, BT549, MCF7, MD-MB231, MD-MB453, MD-MB468 cell lines were cultured in Dulbecco's modified Eagle's medium (Lonza); HCC1954 cell line was cultured in RPMI medium (Gibco). All cells were grown at 37°C and 5% CO2 in the specific growth medium supplemented with 10% fetal bovine serum (FBS), penicillin and streptomycin (100 U/ml). MCF10A cell line was cultured in F12 Dulbecco's modified Eagle's medium (Lonza) supplemented with 20% horse serum, cholera toxin 50 ng/ml (Sigma, C8052), Hydrocortisone 0, 5 μg/ml (Sigma, H0888), Epidermal Growth factor, EGF, 20 ng/ml (Tebu-Bio AF-100-15-B), Human Insuline 0, 01 mg/ml (Roche, 11376497001). Silencing was performed using Lipofectamine RNAiMax (Invitrogen) according to the manufacturer's protocol. We used the following shRNA: siGENOME siRNA Human SETDB1 D-020070-01 20 nmol Dharmacon, ON-TARGET plus SMART pool Human TP63 L-003330-00 20 nmol Dharmacon, Negative control siRNA 20 nmol 1027310 Qiagen. For proteasome inhibition cells were treated for 12 h with MG132 (Sigma), at 20 μM.

### Transfection, plasmids and mutants construction

Transfections were performed using Lipofectamine LTX (Invitrogen), according to the manufacturer's instructions. Human p63 is a pcDNA3.1 expression vector for HA-tagged p63. Primers used to make deletion constructs are listed in Supplementary Table S6. SETDB1 expressing vector is a TrueORF Gold Expression-validated cDNA clones (Origene, RC226620 NM_001145415). Primers used for amplification of SETDB1 ORF and to make deletion constructs are listed in Supplementary Table S5.

### Clonogenic assay

MCF7 and HCC1954 cells were firstly silenced for SETDB1, plated (100, 200, 400, 800 cells) on six-well plates and incubated at 37°C for 11 days changing the medium every three days. Then, cells were fixed and painted with a mixture of 6.0% glutaraldehyde and 0.5% crystal violet for 30′, washed and at last visualized and counted by using ImageJ programme.

### Evaluation of apoptosis and cell cycle

HCC1954 cells were trypsinized, combined with any floating cells present and then washed with PBS. Cells were fixed in 70% cold ethanol, incubated with RNase A for 15 min at 37°C and stained with 50 mg/ml propidium iodide (PI) for 1 h at 37°C. Cell cycle and apoptosis were analysed using a FACS Calibur flow cytometer (BD Biosciences, San Jose, CA, USA). Ten thousand events were evaluated using the Cell Quest (BD) software.

### Western blotting

Immunoblot analysis was performed using whole-cell extracts obtained by lysing the cell pellet with Triton buffer (50 mM Tris-HCl pH 7.5, 250 mM NaCl, 50 mM NaF, 1 mM EDTA 1 pH 8, 0.1% Triton) supplemented with proteases and phosphatases inhibitors. Proteins were resolved on an SDS-10% polyacrylamide gel and blotted onto a Hybond P PVDF membrane (G & E Healthcare). Membranes were blocked with PBST 5% non-fat dry milk, incubated with primary antibodies for 2 h at room temperature, washed and hybridized for 1 h at room temperature using the appropriate horseradish peroxidase-conjugated secondary antibody (rabbit and mouse; BioRad, Hercules, CA, USA). Detection was performed with the ECL chemiluminescence kit (Perkin Elmer, Waltham, MA, USA). The following antibodies were used: anti- β actin (Sigma AC15, dilution 1:50000), anti-SETDB1 (Thermo Scientific 5H6D4, diluition 1:1000), anti-FLAG rabbit (Sigma F7425, diluition 1:1000), anti-HA (Abcam ab130275, diluition 1:1000), anti-p63 BC4A4 mouse (Abcam ab735, diluition 1:200), anti-p63 rabbit (Abcam ab97865, diluition 1:200), anti-p63 4A4 mouse (Sigma P3737, diluition 1:500), anti-p63 3.1 mouse [54, 63] (diluition 1:500), anti-p53 (Santa Cruz, diluition 1:1000), anti-H3K9me3 (Millipore, diluition 1:1000). Supplementary Figures S5–S10 show un-cropped images of western blots.

### Immunoprecipitation

H1299 cells were transiently transfected with 10 μg of total DNA of the indicated mammalian expression plasmids and harvested 24 h after transfection. The cells were then lysed in Triton buffer as described above. After preclearing for 1 h at 4°C, immunoprecipitation was performed by incubating 800 μg of whole-cell protein extracts with an anti-FLAG M2 mouse (Sigma F3165, diluition 1:150) or anti-SETDB1 (Thermo Scientific 5H6D4, diluition 1:150) with rocking at 4°C overnight. The immune complexes were collected by incubation with protein G-sepharose 4 fast flow (G and E Healthcare) for 1 h and washed with Triton buffer. The beads were then resuspended in 25 μl SDS Laemmli sample buffer, subjected to SDS-PAGE (10% polyacrylamide) analysis, and electrotransferred onto PVDF membranes. The membranes were probed with primary antibodies as described above. Same procedures were also used to immunoprecipitate at endogenous level p63 and evaluate SETDB1 in MCF7 and HNEK cell lines.

### Confocal microscopy

Cells were fixed in 4% paraformaldehyde, permeabilized with 0.3% TritonX-100 and blocked with 5% goat serum. Then, they were incubated with an anti-p63 rabbit (Abcam ab97865, diluition 1:100) and an anti-H3K9me3 (Millipore, diluition 1:1000) in PBS containing 5% goat serum for 2 h, followed by incubation with goat anti-mouse and goat anti-rabbit conjugated to AlexaFluor fluorophores 488 and 568 nm, respectively. Nuclei were stained with DAPI and images were obtained using a C1 Nikon microscope and related software.

### Real time and semi quantative PCR analysis

Total RNA from cells was isolated using RNeasy mini kit (Qiagen) following the manufacturer's protocol and it was quantified using a NanoDrop Spectophotometer (Thermo Scientific). RNA was reverse-transcribed using GoScriptTM Reverse Transcription System (Promega) according to manufacturer's protocols. Real time PCR was performed using GoTaq qPCR Mastermix (Promega). The relative expression of each gene was defined from the threshold cycle (Ct), and relative expression levels were calculated by using the 2−ΔΔCt method. The human GAPDH was used as a housekeeping gene for normalization. The sequences of the primers used in this study are indicated in Supplementary Table S3. Semi Quantative PCR analysis was performed using GoTaq G2 Flexi DNA Polymerase (Promega). The human Actin was used as a housekeeping gene for normalization. The sequences of the primers used in this study are indicated in Supplementary Table S3–S6. The primers used to analyse the 90 selected genes by real time-PCR are indicated in Supplementary Table S7.

### Yeast-two hybrid screening

Yeast two-hybrid screening of the Human Breast Tumor Epithelial Cells was performed by the HYBRIGENICS services (http://www.hybrigenics-services.com). The construct used for the screening contains a p63 fragment from aminoacid (aa) 444 to 680 fused to the DNA binding domain of LexA.

### Bioinformatics analysis

By using the cBio Cancer Genomics Portal (available via internet http://www.cbioportal.org) we analysed two datasets of Breast Invasive Carcinoma focusing on SETDB1 amplification and mutations. Additionally, by using GINT database (Gene Interaction survival analysis In Cancer, http://bioprofiling.de) we evaluated breast cancer patients survival of several genes repressed by both p63 and SETDB1.

## SUPPLEMENTARY MATERIALS FIGURES AND TABLES



## Abbreviations

aa: amino acid
HNSCC: head and neck squamous cell carcinomas
TA: transactivation domain
DBD: DNA binding domain
OD: oligomerization domain
SAM: sterile alpha motif
TI: trans inhibitory domain
SID: selected interaction domain
TUD: Tudor domain
MBD: methyl-CpG-binding domain
HMEC: human mammary epithelial cells
HMT: histone methyltransferase
SET: Suppression of variegation, Enhancer of zeste, Trithorax.
