# Supplementary material for: Setdb1, a novel interactor of ΔNp63, is involved in breast tumorigenesis
Source: Oncotarget. 2016 Jan 31;7(20):28836–48. doi: 10.18632/oncotarget.7089 (PMC5045360; doi:10.18632/oncotarget.7089)
Supplement: Supplementary file 1 [file oncotarget-07-28836-s001.pdf]

Setdb1, a novel interactor of ΔNp63, is involved in breast tumorigenesis

Supplementary Materials

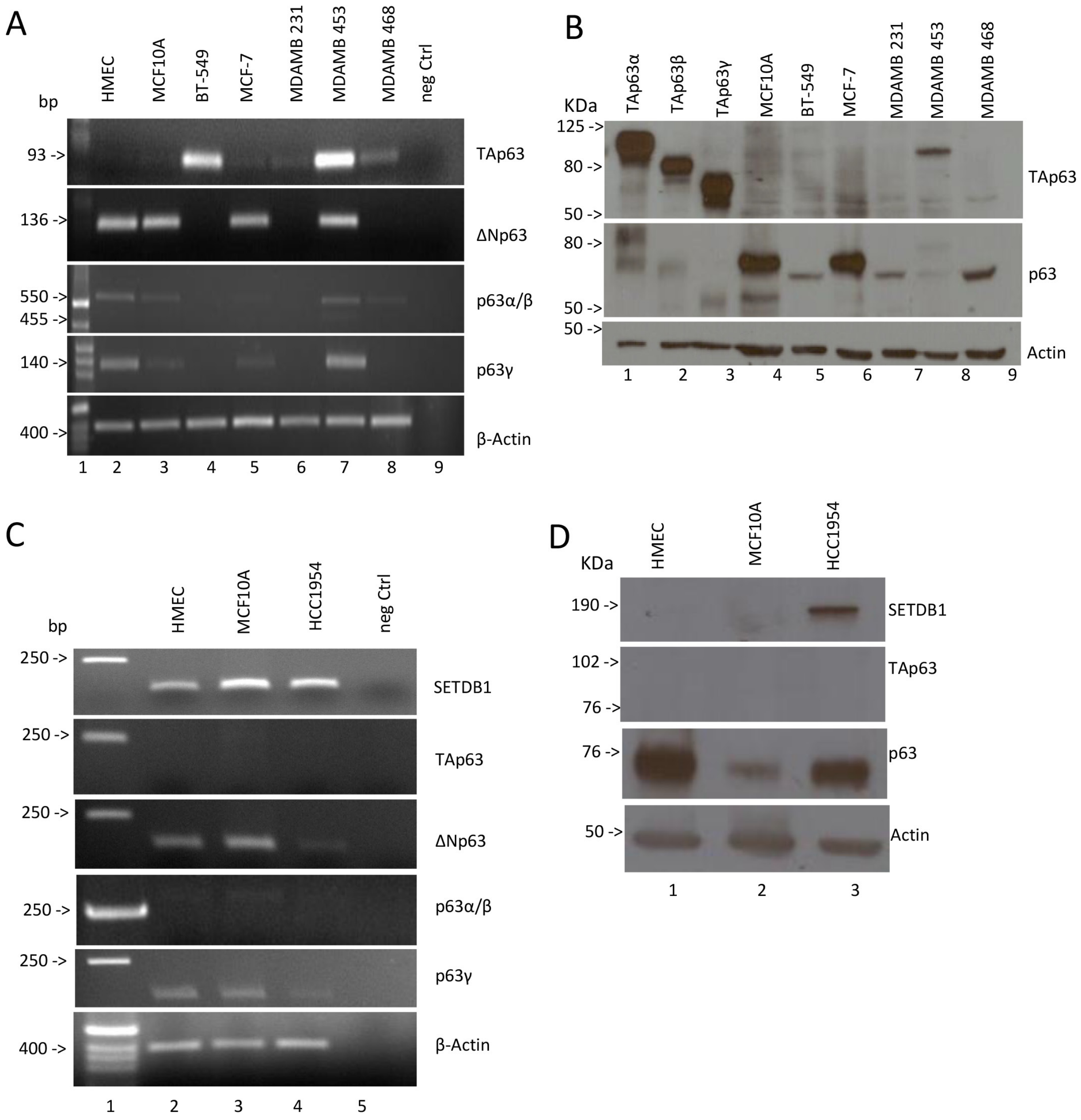

Supplementary Figure S1: Expression analysis of p63 isoforms in breast cancer cell lines. (**A**) Semi-quantitative reverse transcriptase analysis of p63 isoforms mRNA levels in different breast cancer cell lines (basal type: BT-549, MDM-AMB231, MDM-AMB468; luminal type: MCF-7, MDM-AMB453). Human Mammary Epithelial Cells (HMEC) and MCF-10A have been used as normal primary and immortalized breast epithelial cells). β-Actin is shown as loading control. One representative experiment of three is shown. (**B**) Western blot analysis of p63 isoforms in the breast cell lines stated above. β-Actin is shown as loading control. One representative experiment of three is shown. HEK-293 cells overexpressing TAp63α, TAp63β and TAp63γ have been used as positive controls. (**C**) Semi-quantitative reverse transcriptase analysis of p63 isoforms and SETDB1 mRNA levels in HCC1954 basal breast cell line. Human Mammary Epithelial Cells (HMEC) and MCF-10A have been used normal primary and immortalized breast epithelial cells). β-Actin is shown as loading control. One representative experiments of three is shown. (**D**) Western blot analysis of p63 isoforms and SETDB1 in the breast cell lines stated above. β-Actin is shown as loading control. One representative experiment of three is shown.

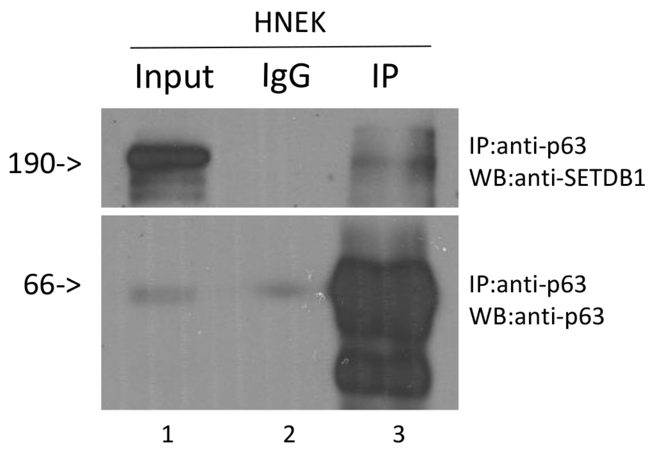

Supplementary Figure S2: Immunoprecipitation of endogenous p63 with endogenous SETDB1. Human normal epithelial keratinocytes (HNEK) cells extracts were immunoprecipitated with anti-p63 antibody and western blot analysis (lane 3) with anti-SETDB1 antibody (upper panel) and anti-p63 antibody (lower panel). The aliquots of total cell extracts from unprocessed cells (lane 1) and IgG, used as negative control (lane 2), were also loaded was performed.

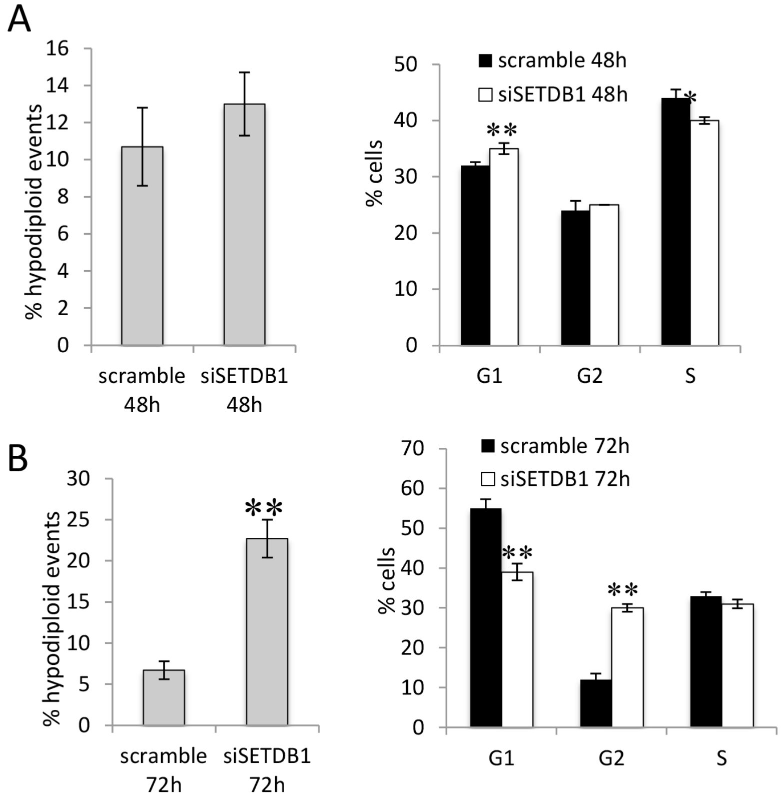

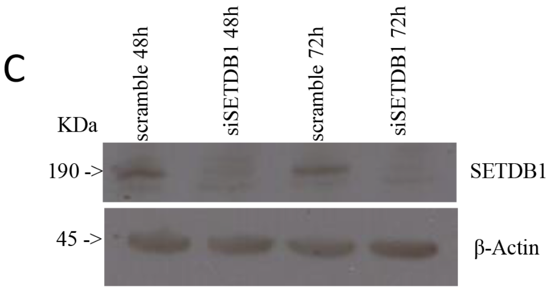

Supplementary Figure S3: Depletion of SETDB1 induces cell death and decreases proliferation. Apoptosis and cell cycle analysis of HCC1954 breast cancer cell line silenced for SETDB1 and collected at 48 hours (**A**) and 72 hours (**B**) are shown. Silencing of SETDB1 causes an increase of hypodiploid events at both 48 hours and 72 hours, with a significative result only at 72 hours. Cell cycle analysis shows that SETDB1 depletion causes a significative increase of G1, a significative decrease of S but no variation in G2 phase at 48 h (A). 72 h silencing causes a significative decrease of G1, a significative increase of G2 but no variation in S phase (B). Silencing controls are shown in figure C (**C**).

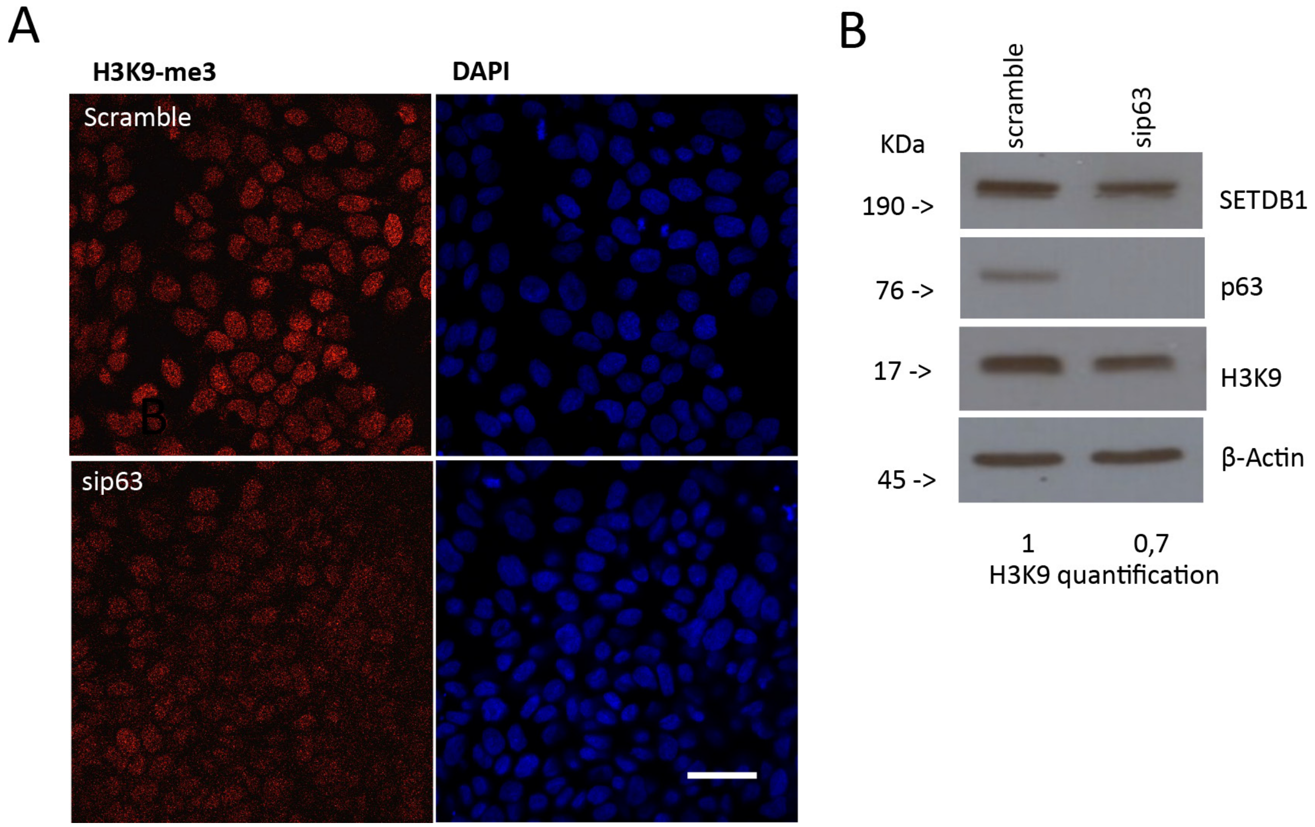

Supplementary Figure S4: p63 knock-down decreases H3K9-me3 deposition. Immunofluorescence (**A**) and Western Blotting (**B**) of HCC1954 showing the decrease of H3K9-me3 after p63 silencing. Western Blotting quantification of H3K9-me3 levels is also shown.

Figure 1A

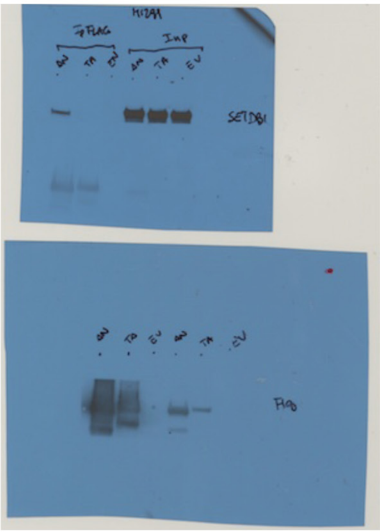

Figure 1B

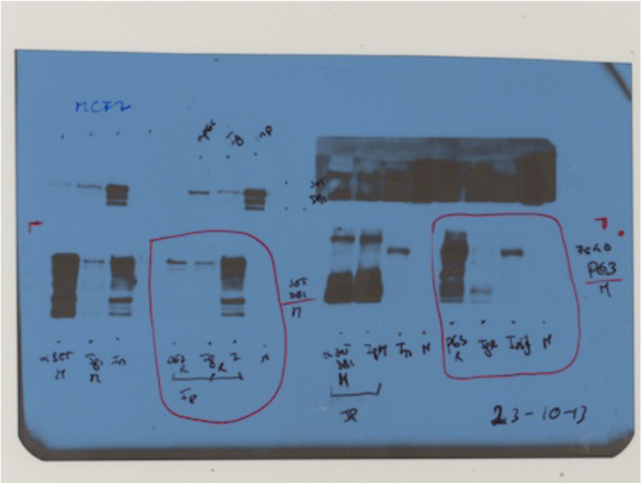

Figure 1D

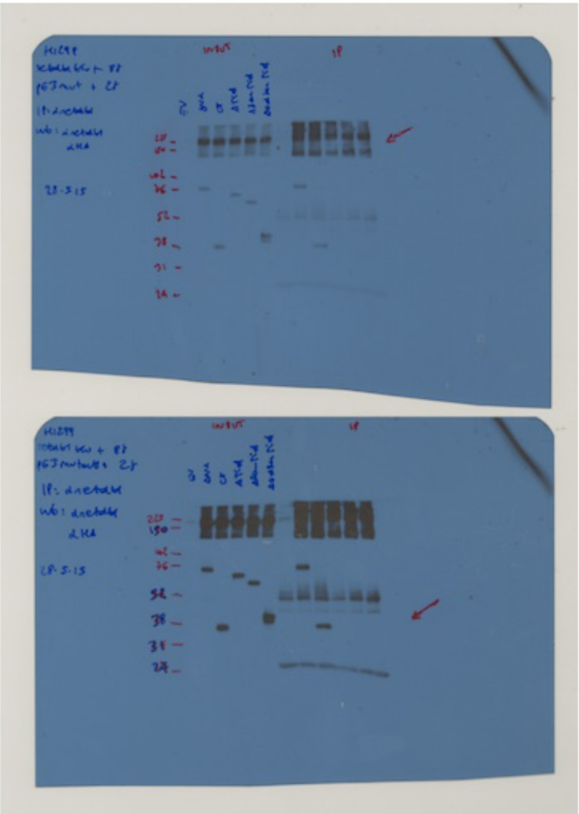

Figure 1F

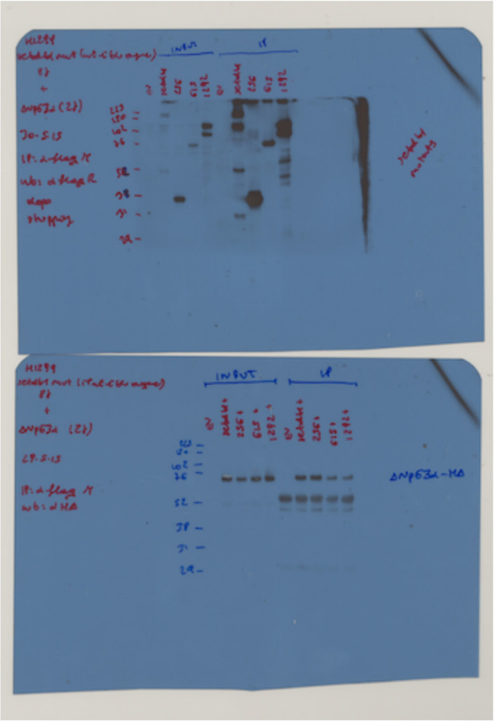

Supplementary Figure S5: Uncropped images. Uncropped images of scanned films for Figure 1 are shown.

Figure 2A: w/blot SETDB1, 72 and 96 h

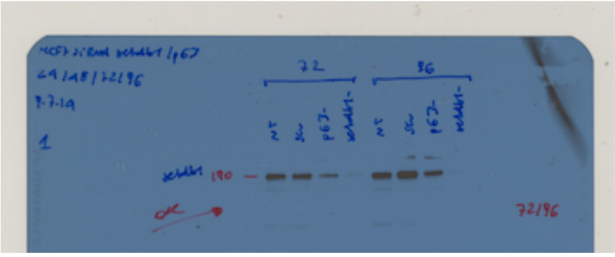

Figure 2A: w/blot SETDB1, 48 h

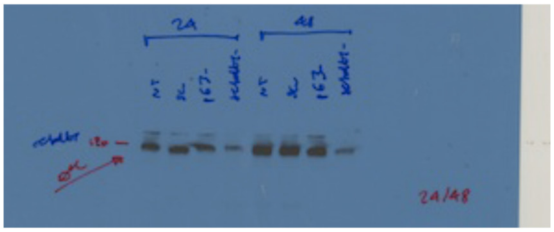

Figure 2A: w/blot p63, 48, 72 and 96 h

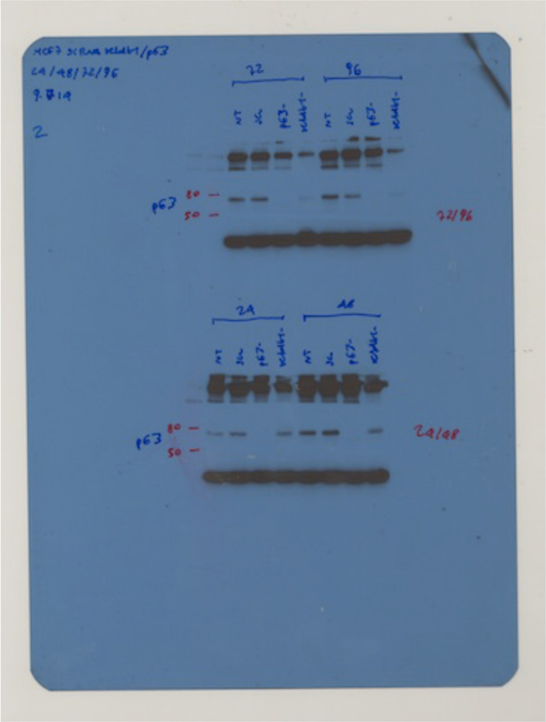

Figure 2A: w/blot beta-actin 48, 72 and 96h

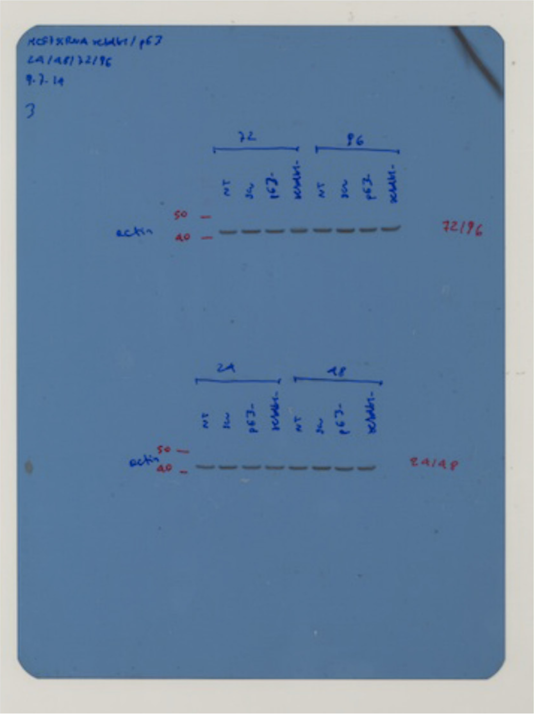

Supplementary Figure S6: Uncropped images. Uncropped images of scanned films for Figure 2A are shown.

Figure 2D: w/blot SETDB1 and p63

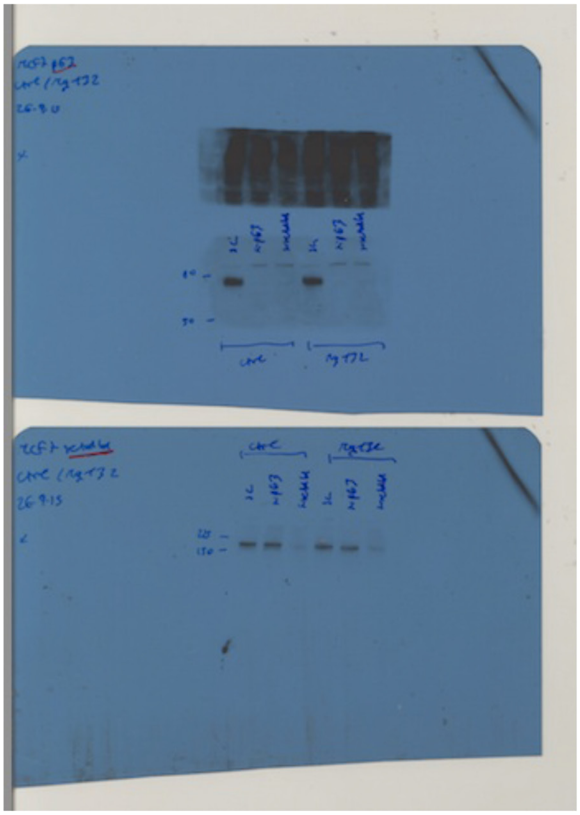

Figure 2D: w/blot p53 and beta actin

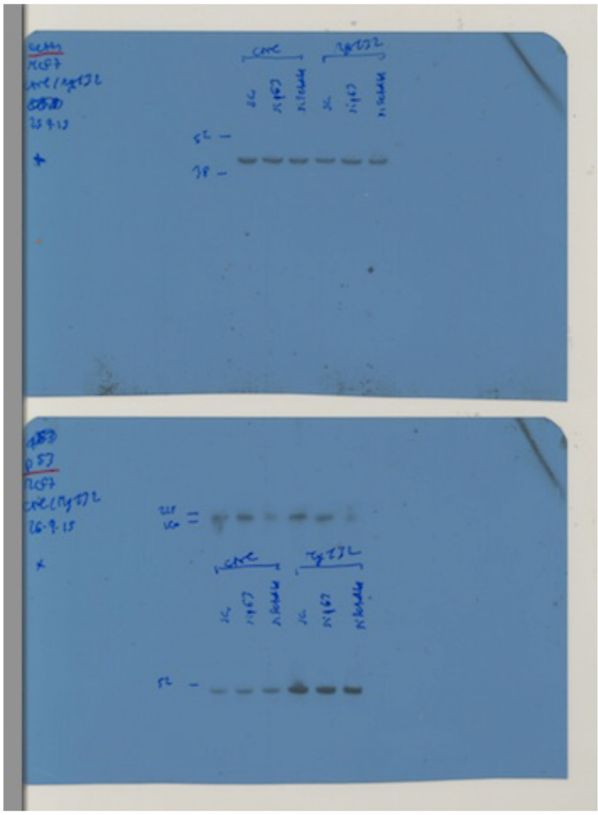

Supplementary Figure S7: Uncropped images. Uncropped images of scanned films for Figure 2D are shown.

Figure 3B: w/blot SETDB1 and beta-actin

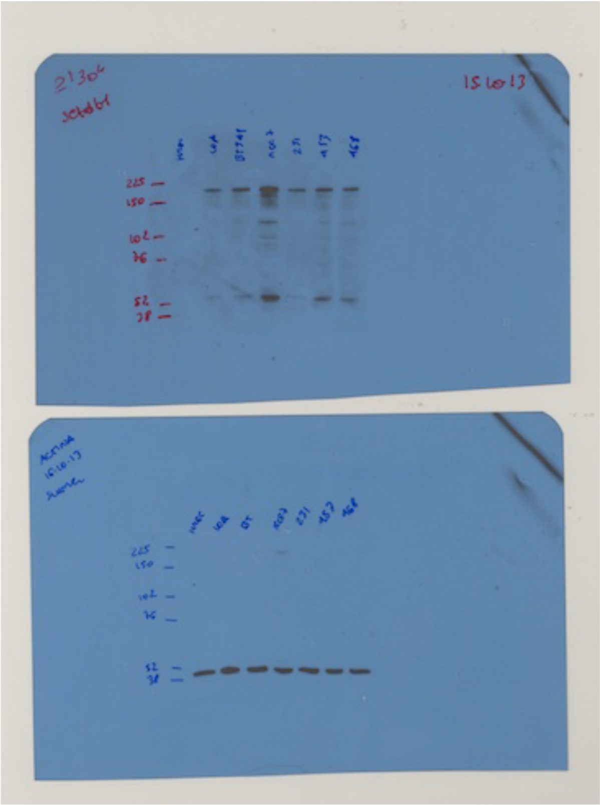

Supplementary Figure S8: Uncropped images. Uncropped images of scanned films for Figure 3B are shown.

Figure S1: w/blot SETDB1 and p63

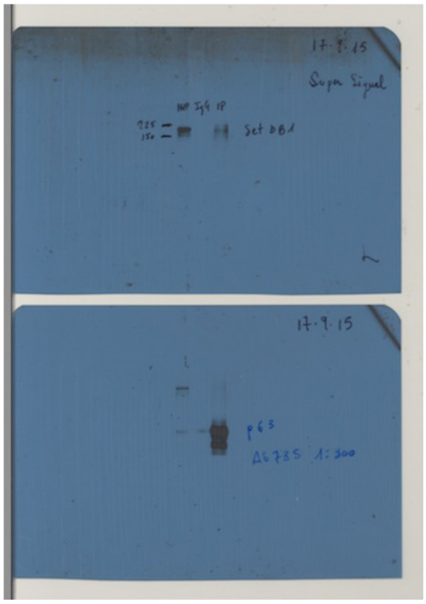

Figure S2B: w/blot p63 and beta actin

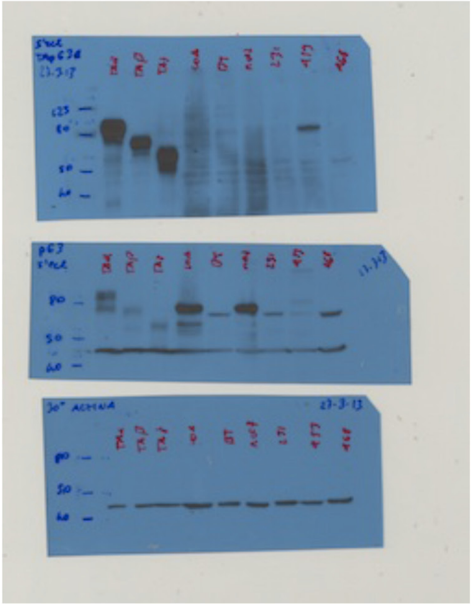

Figure S2D: w/blot p63 and beta actin

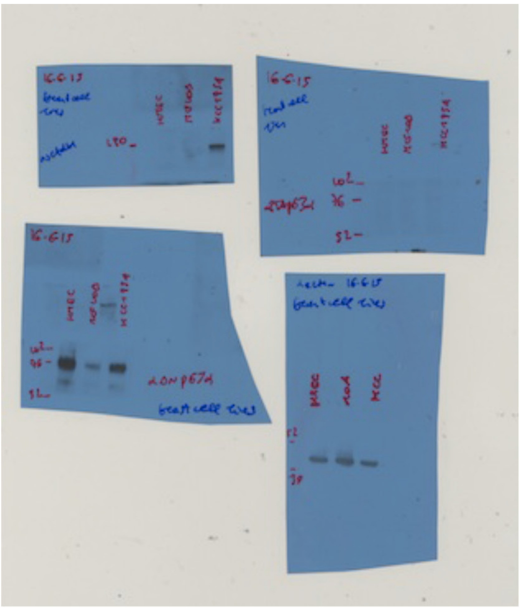

Figure S3C: w/blot SETDB1
and beta-actin

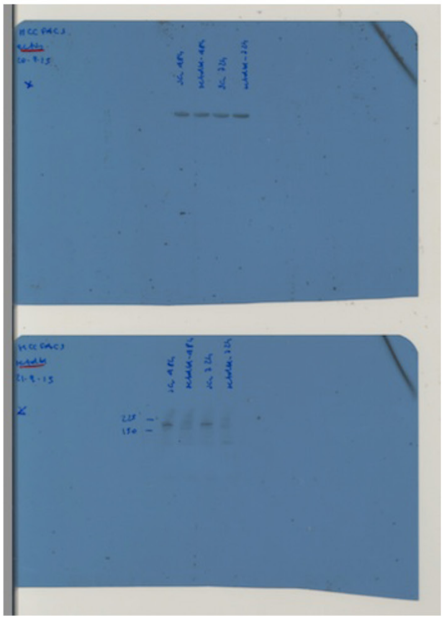

Supplementary Figure S9:Uncropped images. Uncropped images of scanned films for Figure S1, S2A, S2B, S3C are shown.

Figure S4B: w/blot SETDB1, p63, actin and H3K9me3

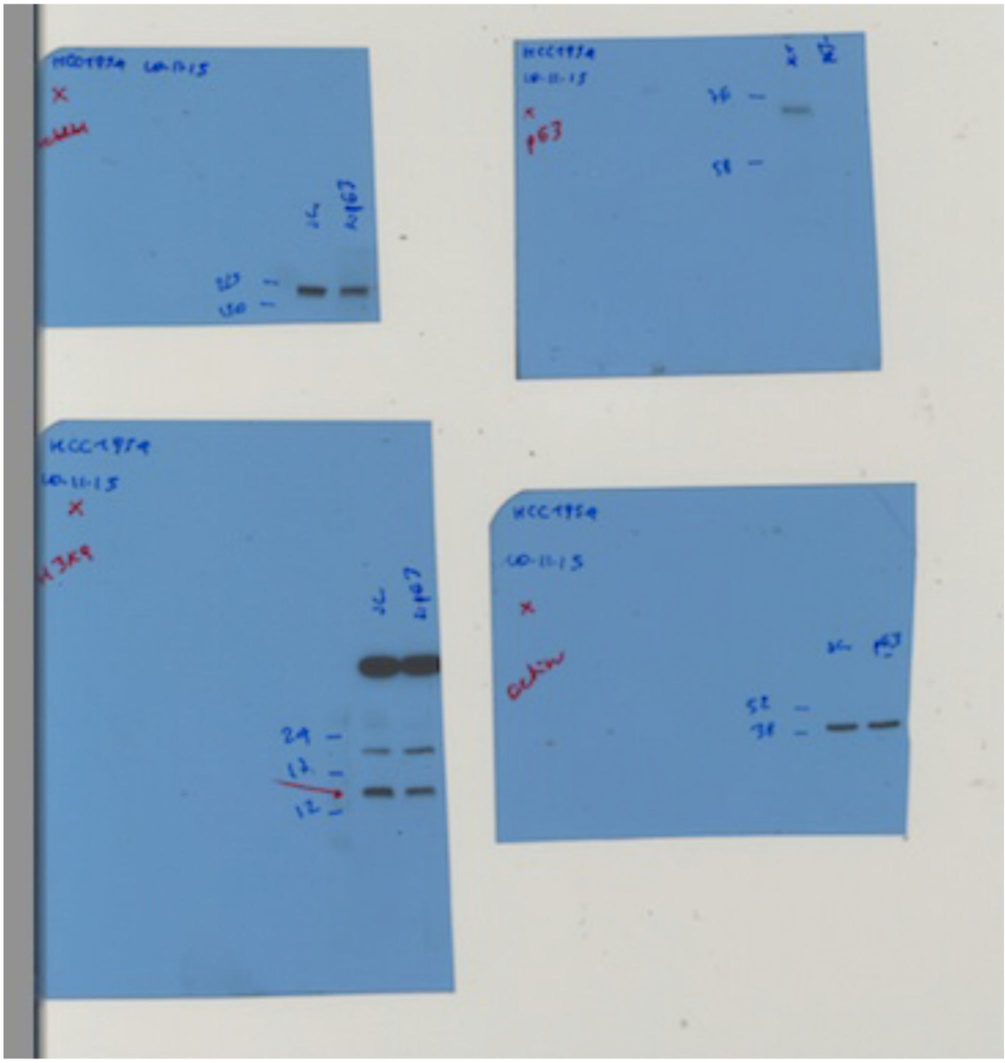

Supplementary Figure S10: Uncropped images. Uncropped images of scanned films for Figure S4B are shown.

Supplementary Table S1: genes repressed by p63 and SETDB1

Gene Symbol	sip63	siSETDB1
	Fold/Ctrl	Fold/Ctrl
ABHD4	2, 35	1, 75
ACCA2	3, 34	–
ACSL1	2, 44	–
ACTA2	2, 81	2, 45
ADCY9	3, 18	2, 08
ALDH3B2	3, 97	–
ANKRD22	2, 07	1, 46
ANXA9	6, 05	1, 62
ARRB1	3, 02	–
ASS1	2, 50	1, 53
CASZ1	2, 71	–
CD74	2, 07	–
CGN	3, 00	1, 62
CITED2	2, 07	1, 87
CLIC3	2, 26	–
CRIP2	9, 63	1,86
CTSO	4, 28	–
CYP4B1	2, 78	–
DUSP8	3, 86	1, 65
EDN2	3, 26	–
EFNA1	2, 70	–
ELF3	3, 01	1, 46
ERBB3	2, 22	1, 85
FBXO32	2, 16	–
FER1L6	3, 35	3, 10
GAB2	2, 81	1, 74
GABARAP	2, 39	–
GBP2	4, 23	1, 55
GGT6	6, 91	1, 66
GLIPR2	2, 25	–
GPR110	3, 89	–
HCP5	4, 33	1, 65
HYAL1	3, 18	–
IGFBP3	2, 87	1, 30
KLF4	2, 48	1, 47
LBH	3, 36	1, 72
ME3	3, 43	–
MEIS2	4, 82	–
MFAP2	3, 18	4, 97
MTMR11	3, 39	1, 63
MUC1	5, 05	1, 59
ORAI2	2, 29	–
PDZK1IP1	2, 63	1, 40

PPL	3, 24	1, 55
SAMD9L	2, 19	1, 97
SCNN1A	3, 74	1, 50
SEPT6	2, 28	1, 46
SLC2A3	2, 82	2, 74
SLC28A3	4, 09	–
SLC22A18	3, 64	–
SULT2B1	2, 22	–
TJP3	4, 72	
UPK2	2, 29	1, 38
VLDLR	3, 04	–
ZNF837	2, 34	–

Supplementary Table S2: Cancer survival analysis for identified gene list

**Datasets**	**Positive genes**	
	**Gene Symbol**	***p*-value**
**GSE25065**	ANXA9	0.000339
	CRIP2	0.000339
	ERBB3	0.00307
	CITED2	0.00777
**GSE17705**	ADCY9	0.000846
	ACTA2	0.00194
	NPAS2	0.00381
	PPL	0.00895
**GSE25055**	ADCY9	1.81e–05
	ANXA9	0.000121
	ERBB3	0.000188
	SCNN1A	0.0011
	CRIP2	0.00123
**GSE31448**	SEPT6	0.00268
	GBP2	0.00357
	ERBB3	0.00507
**GSE30682**	ANXA9	5.44e–05
	SCNN1A	0.000995

1) PPISURV: a novel bioinformatics tool for uncovering the hidden role of specific genes in cancer survival outcome. Antonov AV, Krestyaninova M, Knight RA, Rodchenkov I, Melino G, Barlev NA. Oncogene. 2014 Mar 27;33(13):1621-8.
2) BioProfiling.de: analytical web portal for high-throughput cell biology. Antonov AV. Nucl. Acids Res. (2011) 39.

Supplementary Table S3: List of the primers used for Real Time PCR

Name	Primer sequences
hSETDB1	F: 5′ – TCGGGTGGTCGCCAAA – 3′<br>R: 5′ – TCAGCTACAATGCCAGCATAGAG – 3′
hΔNp63	F: 5′ – GAAGAAAGGACAGCAGCAT – 3′<br>R: 5′ – GGGACTGGTGGACGAGGAG – 3′
hGAPDH	F: 5′ – AGCCACATCGCTCAGACAC – 3′<br>R: 5′ – GCCCAATACGACCAAATCC – 3′

Supplementary Table S4: List of the primers used for Semi Quantative PCR

Name	Primer sequence
hSETDB1	F: 5′ – TTGCTTCCCCTTCCCTCTTTCACG – 3′<br>R: 5′ – TTTCACCAACCAGACCCCAGACTC – 3′
hTAp63	F: 5′ – TCAGAAGATGGTGCGACAAAC – 3′<br>R: 5′ – GTTCAGGAGCCCCAGGTTCG – 3′
hΔNp63	F: 5′ – GAAGAAAGGACAGCAGCAT – 3′<br>R: 5′ – GGGACTGGTGGACGAGGAG – 3′
hp63α/β	F: 5′ – TCTTTTGCCACCAACATCC – 3′<br>R: 5′ – AACATCCCTGTTGCTGAAAC – 3′
hp63γ	F: 5′ – CGTCAGAACACACATGGTATCCAGAT – 3′<br>R: 5′ – GGGTACACTGATCGGTTTGGG – 3′
hActin	F: 5′ – CTGGCACCACACCTTCTACAATG – 3′<br>R: 5′ – AATGTCACGCACGATTTCCCG – 3′

Supplementary Table S5: List of the primers used for the amplification of SETDB1 ORF and in the deletion mutants construction

Name	Primer sequence
Flag-SETDB1 wt	F: 5′ – GCGGTACCTTCTTCCCTTCCTGGGTGCAT – 3′<br>R: 5′ – GGGCGGCCGCCTAAAGAAGACGTCCTCT – 3′
Flag 1–256 aa	F: 5′ – GCGGTACCTTCTTCCCTTCCTGGGTGCAT – 3′<br>R: 5′ – GGGCGGCCGCCTAGTCAGCAGGAGGGT – 3′
Flag 1–615 aa	F: 5′ – GCGGTACCTTCTTCCCTTCCTGGGTGCAT – 3′<br>R: 5′ – GGGCGGCCGCCTAACGCCGGGCTGTCAT – 3′
Flag 528–1307 aa	R: 5′ – GGGCGGCCGCCTAAAGAAGACGTCCTCT – 3′<br>F: 5′ – GCGGTACCTTCACCTTTAGGCTCCACAG – 3′

Supplementary Table S6: List of the primers used for the amplification of ΔNp63 ORF and in the deletion mutants construction

Name	Primer sequence
HA-ΔNp63 wt	F: 5′ – CGGGGATCCTTGTACCTGGAAAACAATGCCC – 3′<br>R: 5′ – CCGGCTCGAGTCACTCCCCCTCCTCTTTGATGCGC – 3′
HA-CT	F: 5′ – CGGGGATCCTACAAAGAACGGTGATGGTACG– 3′<br>R: 5′ – CCGGCTCGAGTCACTCCCCCTCCTCTTTGATGCGC– 3′
HA-ΔTID	F: 5′ - CGGGGATCCTTGTACCTGGAAAACAATGCCC – 3′<br>R: 5′ – CCGGCTCGAGTCAGAGCTGCCGGTGGTCCAGGATGC– 3′
HA-ΔSAM-TID	F: 5′ – CGGGGATCCTTGTACCTGGAAAACAATGCCC – 3′<br>R: 5′ - CCGGCTCGAGTCATGGGGGTGTGCAGTGGGAGG– 3′
HA-NT	F: 5′ – CGGGGATCCTTGTACCTGGAAAACAATGCCC – 3′<br>R: 5′ – CCGGCTCGAGTCACTGTCCGAAACTTGCTGCTTTCTGATGC– 3′
